# Zerumbone liquid crystalline nanoparticles protect against oxidative stress, inflammation and senescence induced by cigarette smoke extract *in vitro*

**DOI:** 10.1007/s00210-023-02760-7

**Published:** 2023-10-18

**Authors:** Keshav Raj Paudel, Dvya Delilaa Clarence, Nisha Panth, Bikash Manandhar, Gabriele De Rubis, Hari Prasad Devkota, Gaurav Gupta, Flavia C. Zacconi, Kylie A. Williams, Lisa G. Pont, Sachin Kumar Singh, Majid Ebrahimi Warkiani, Jon Adams, Ronan MacLoughlin, Brian G. Oliver, Dinesh Kumar Chellappan, Philip Michael Hansbro, Kamal Dua

**Affiliations:** 1 Centre of Inflammation, Centenary Institute and University of Technology Sydney, Faculty of Science, School of Life Sciences, Sydney, NSW 2007 Australia; 2 School of Postgraduate Studies, International Medical University (IMU), 57000 Kuala Lumpur, Malaysia; 3 Discipline of Pharmacy, Graduate School of Health, University of Technology Sydney, Sydney, NSW 2007 Australia; 4 Faculty of Health, Australian Research Centre in Complementary and Integrative Medicine, University of Technology Sydney, Ultimo, NSW 2007 Australia; 5 Graduate School of Pharmaceutical Sciences, Kumamoto University, 5-1 Oe-honmachi, Chuo-ku, Kumamoto City, Kumamoto, 862-0973 Japan; 6 Program for Leading Graduate Schools, Health Life Science: Interdisciplinary and Glocal Oriented (HIGO) Program, 5-1 Oe-honmachi, Chuo-ku, Kumamoto, 862-0973 Japan; 7 Center for Global Health Research, Saveetha Institute of Medical and Technical Sciences, Saveetha University, Chennai, Tamil Nadu 602105 India; 8 School of Pharmacy, Graphic Era Hill University, Dehradun, Uttarakhand 248007 India; 9 School of Pharmacy, Suresh Gyan Vihar University, Jagatpura, Mahal Road, Jaipur, 302017 India; 10 Departamento de Química Orgánica, Facultad de Química y de Farmacia, Pontificia Universidad Católica de Chile, Av. Vicuña Mackenna 4860, 7820436 Santiago, Macul Chile; 11 Centro de Investigación en Nanotecnología y Materiales Avanzados, CIEN-UC, Pontificia Universidad Católica de Chile, Av. Vicuña Mackenna 4860, Macul, 7820436 Santiago, Chile; 12 Institute for Biological and Medical Engineering, Schools of Engineering, Medicine and Biological Sciences, Pontificia Universidad Católica de Chile, Santiago, Chile; 13 School of Pharmaceutical Sciences, Lovely Professional University, Jalandhar-Delhi GT Road, Phagwara, Punjab 144411 India; 14 School of Biomedical Engineering, University of Technology Sydney, Sydney, NSW 2007 Australia; 15 Institute for Biomedical Materials and Devices, Faculty of Science, University of Technology Sydney, Sydney, NSW 2007 Australia; 16 Aerogen, IDA Business Park, Dangan, Galway, H91 HE94, Ireland; 17 School of Pharmacy & Biomolecular Sciences, Royal College of Surgeons in Ireland, Dublin, D02 YN77, Ireland; 18 School of Pharmacy & Pharmaceutical Sciences, Trinity College, Dublin, D02 PN40, Ireland; 19 Woolcock Institute of Medical Research, University of Sydney, Sydney, New South Wales Australia; 20 School of Life Sciences, University of Technology Sydney, Ultimo, NSW 2007 Australia; 21 Department of Life Sciences, School of Pharmacy, International Medical University, Bukit Jalil, 57000 Kuala Lumpur, Malaysia

**Keywords:** Zerumbone, Liquid crystalline nanoparticles, Monoolein, P407, Anti-inflammatory, Antioxidant

## Abstract

The purpose of this study was to evaluate the potential of zerumbone-loaded liquid crystalline nanoparticles (ZER-LCNs) in the protection of broncho-epithelial cells and alveolar macrophages against oxidative stress, inflammation and senescence induced by cigarette smoke extract *in vitro*. The effect of the treatment of ZER-LCNs on *in vitro* cell models of cigarette smoke extract (CSE)-treated mouse RAW264.7 and human BCi-NS1.1 basal epithelial cell lines was evaluated for their anti-inflammatory, antioxidant and anti-senescence activities using colorimetric and fluorescence-based assays, fluorescence imaging, RT-qPCR and proteome profiler kit. The ZER-LCNs successfully reduced the expression of pro-inflammatory markers including *Il-6*, *Il-1β* and *Tnf-α*, as well as the production of nitric oxide in RAW 264.7 cells. Additionally, ZER-LCNs successfully inhibited oxidative stress through reduction of reactive oxygen species (ROS) levels and regulation of genes, namely *GPX2* and *GCLC* in BCi-NS1.1 cells. Anti-senescence activity of ZER-LCNs was also observed in BCi-NS1.1 cells, with significant reductions in the expression of *SIRT1*, *CDKN1A* and *CDKN2A*. This study demonstrates strong *in vitro* anti-inflammatory, antioxidative and anti-senescence activities of ZER-LCNs paving the path for this formulation to be translated into a promising therapeutic agent for chronic respiratory inflammatory conditions including COPD and asthma.

## Introduction

Inflammatory lung diseases represent a common cause of illness for many individuals, and they are mainly caused by toxins, chemicals, allergens and foreign antigens. One of the main inflammatory lung diseases is chronic obstructive pulmonary disease (COPD), a slow-developing and incurable ailment with complex multifactorial aetiology. COPD is estimated to cause the third largest number of deaths worldwide, with an estimated 3 million casualties every year (Eapen et al., 2017). COPD affects individuals aged 40 years or older, particularly cigarette smokers, who are exposed to over 4500 harmful chemicals contained in cigarette smoke that contribute to the pathogenesis of COPD (Hikichi et al., 2019, Lugg et al., 2022). Among the harmful substances contained in cigarette smoke, there are also oxygen radicals, which are present in an estimated number of 10^15^ radicals per cigarette puff (Rahman and MacNee, 1996). The main features of COPD are airway inflammation, remodelling and mucus retention (Vogelmeier et al., 2017), leading to limited airflow into the lungs, accompanied by wheezing, cough, difficulty in breathing, sputum production, dyspnoea, increased chest wall diameter and progressive and irreversible airway hyperresponsiveness.

Although the exact pathogenesis of COPD is not fully understood, common processes involved in COPD pathogenesis include airway inflammation, oxidative stress, impaired immunity (both innate and adaptive) and imbalance between protease and anti-protease (Wang et al., 2020). The main inflammatory cytokines released upon cigarette smoking include IL (interleukin)-6, IL-1β, IL-8 and CXCL-8, released by broncho-epithelial cells, and tumour necrosis factor-alpha (TNF-α), nitric oxide (NO) and granulocyte macrophage-colony stimulating factor (GM-CSF), which are released by the macrophages in the alveolar space (Paudel et al., 2022c). Besides the release of cytokines and chemokines, cigarette smoke causes apoptosis, senescence and oxidative stress (Paudel et al., 2022c). Oxidative stress is caused by excessive formation of reactive oxygen species (ROS). ROS also elicit pro-inflammatory effects that tend to worsen the inflammation in COPD through the activation of several pro-inflammatory transcriptional pathways (King, 2015). Furthermore, oxidative stress can damage DNA, inhibit tissue repair (Nakamaru et al., 2009), accelerate aging and cause senescence in the lung parenchyma (Mercado et al., 2015).

Although conventional COPD treatment strategies of bronchodilators, corticosteroids, steroids and antibiotics can relieve the symptoms, these treatments present many side effects including headaches, insomnia, palpitations, weight gain, mood swings and osteoporosis (Bollmeier and Hartmann, 2020). Hence, there is an unmet need to develop novel therapeutic approaches that can treat and prevent the worsening conditions of COPD with minimal side effects. A therapeutic treatment with high potency will be effective at lower doses and minimise adverse effects.

**Fig. 1:**
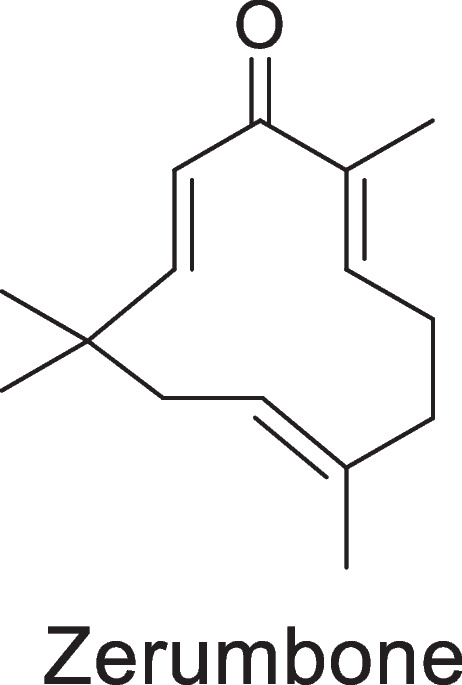
Chemical structure of zerumbone (C_15_H_22_O) as depicted on ChemDraw

Zerumbone (ZER) is a crystalline, monocyclic, naturally occurring phytochemical compound first isolated in 1960 from the essential oil of the traditional plant known as *Zingiber zerumbet.* Historically, this plant has been used to treat various illnesses, including inflammation, fever, asthma, skin diseases, diabetes and many others (Girisa et al., 2019). The structure of ZER (2,6,9,9-tetramethyl-(2E,6E,10E)-cycloundeca-2,6,10-trien-1-one) (Girisa et al., 2019) is shown in Fig. 1. ZER is highly lipophilic, insoluble in water (1.296 mg/L at 25 °C) (Hall et al., 1981) but soluble in solvents such as ethanol. ZER possesses many beneficial activities, including anti-inflammatory (Su et al., 2021), antibacterial (Moreira da Silva et al., 2018), antioxidant (Sidahmed et al., 2015), antipyretic, hepatoprotective (Hamid et al., 2018), antiproliferative (Ghasemzadeh et al., 2017) and anticancer activity (Rahman et al., 2014). Mechanistically, ZER exerts its anti-inflammatory activity by suppressing the production of NO, IL-6, IL-1β, prostaglandin E2 (PSGT2), as well as by decreasing the activity of inducible NO synthase (iNOS), COX-2 and NF-_K_B, which are key inflammatory factors in COPD (Su et al., 2021). The antioxidant activity of ZER is exerted through an increase in glutathione (GSH) levels as well as via the inhibition of thiobarbituric acid reactive substance (TBARS), which are directly related to peroxidation levels in the body (Sidahmed et al., 2015). Despite the therapeutic potential of ZER against inflammatory lung diseases, its poor water solubility translates into poor absorption, reduced oral bioavailability and limited targeting to tissues and organs of interest (Md et al., 2018, Albaayit et al., 2020), limiting its therapeutic efficacy.

The use of nanoparticle drug delivery system has potential to improve the solubility of poorly soluble drugs, simultaneously reducing systemic toxicity and increasing pharmacodynamic action (Mu€ller et al., 2006). Among many available classes of nanocarriers, liquid crystalline nanoparticles (LCNs) have recently gained notable attention due to their remarkable ability to improve drug stability and bioavailability, minimising toxicity (Mo et al., 2017) and allowing modified drug release and site-specific drug delivery (Cerpnjak et al., 2013) as well as high flexibility in terms of drug loading and holding, resulting in modulated drug release and improved long-term stability (Müller et al., 2002). Furthermore, LCNs can encapsulate drugs with different physical properties including hydrophilic, hydrophobic or even amphiphilic, tremendously improving the drug development process. They also have self-assembling properties and can encapsulate drugs with various types of physical properties including hydrophilic, hydrophilic or even amphiphilic which can be very beneficial in the drug development process (Jain et al., 2012).

In this study, we encapsulated ZER in LCNs and tested anti-inflammatory, antioxidant and anti-senescence activity of this novel ZER-LCN formulation on an *in vitro* models of cigarette smoke extract (CSE)-induced oxidative stress, senescence and inflammation in mouse RAW264.7 cells and human BCi-NS1.1 basal epithelial cells. The formulation showed potent anti-inflammatory, antioxidant and anti-senescence activities *in vitro*, by decreasing the expression of many molecular markers of these processes, often to a higher extent as compared to free ZER.

## Materials and methods

### Chemicals and reagents

ZER (MW218.3) was purchased from Funakoshi Co. Ltd. (originally produced by Adipogen Life Science, Japan), monoolein (MO, 1-oleoyl-rac-glycerol, MW 356.55 g/mol, purity 99.5%), poloxamer 407 (P407) and phosphate buffered saline (PBS) containing 137 mM sodium chloride, 2.7 mM potassium chloride and 10 mM phosphate buffer were purchased from Merck (Kenilworth, NJ, USA). MTT (3-[4,5-dimethylthiazol-2-yl]-2,5- diphenyl tetrazolium bromide), dimethyl sulphoxide (DMSO) and dichloro dihydrofluorescein diacetate (DCF-DA) were purchased from Merck. The Griess reagent kit (G7921) used for nitrite quantification was procured from Thermo-Fisher Scientific, Waltham, MA, USA. Rabbit anti-p21 antibody (2947S) and rabbit anti-p16 antibody (18769S) were purchased from Cell Signalling Technology, Danvers, MA, USA. Beta galactosidase staining (X-gal) kit (ab102534) and goat anti-rabbit Alexa Fluor 647 (ab150079) and goat anti-rabbit Alexa Fluor 488 (ab150077)-conjugated antibody were purchased from Abcam, Cambridge, UK. All the solvents and reagents used in the study were of analytical research grade.

### Cell culture

For the *in vitro* experiments, minimally immortalised human airway epithelium derived basal cells (BCi-NS1.1) were obtained from R. G. Crystal (Weill Cornell Medical College, New York, USA). RAW 264.7cells were purchased from ATCC, USA. BCi-NS1.1 cells were grown as submerged culture on a bronchial epithelial basal media with growth supplement (Lonza) and RAW264.7 cells were grown in Dulbecco’s Modified Eagle’s Medium (DMEM). Culture media were supplemented with 5–10% foetal bovine serum, 1% antibiotic mix (penicillin and streptomycin) in humidified 5% CO_2_ incubator. The cells were regularly checked for mycoplasma contamination and all experiments were conducted using only mycoplasma-negative cells. ZER pure compound was dissolved in DMSO and treated to cells with final concentration of DMSO not exceeding 0.01%.

### Preparation of cigarette-smoke extract (CSE)

For the preparation of CSE, reference cigarettes 3R4F were procured from Kentucky University, USA. The CSE was prepared as reported in previous studies by our research team (Paudel et al., 2022b, De Rubis et al., 2023, Malyla et al., 2023). Briefly, one cigarette was burned, and the cigarette smoke produced was bubbled through 10 mL of PBS, producing the 100% CSE which was then passed through 0.22 μM filter and then diluted to a final concentration of 5% CSE. In order to achieve uniformity, the freshly prepared CSE was used to treat the cells no later than 30 min after preparation.

### Preparation of ZER-LCNs

ZER-LCNs were prepared following the same protocol as described previously for other similar formulations (Paudel et al., 2020, Wadhwa et al., 2021, Alnuqaydan et al., 2022, Paudel et al., 2022a).

### Cell-viability assay

The MTT colorimetric assay was used to determine the toxicity caused by CSE and ZER-LCNs on the BCi-NS1.1 and RAW 264.7 cells. For each cell line, cells were seeded separately at a density of 10,000 cells/well in a 96-well plate. Cells were then incubated overnight at 37 °C to allow attachment. Then, cells were pre-treated with or without pure ZER or ZER-LCNs (at final concentrations of 2.5, 5, 10 or 25 μM) for 1 h, followed by exposure to 5% CSE for 24 h. After 24 h, 10 μL of the MTT solution (5 mg/mL stock) was added to each well separately and incubated for another 4 h. The culture media was then taken out and the remaining formazan crystals that were produced by the enzymatic activity of the living cells on MTT were dissolved with 100 μL of the DMSO. Using a microplate reader (POLARstar Omega, purchased through BMG LABTECH Pty. Ltd., Victoria, Australia) at a wavelength of 540 nm, the absorbance of the purple-coloured product was measured. Following this, the viability of the control cells was standardised to 100% and then the viability for cells treated with ZER or ZER-LCNs and 5% CSE was calculated.

### Measurement of total cellular reactive oxygen species

#### Measurement with fluorescence plate reader

RAW 264.7 cells were seeded in a black 96-well plate and incubated overnight prior to the treatment. The cells were pre-treated with pure ZER or ZER-LCNs (final concentrations of 5 and 10 μM) for 1 h, followed by treatment with 5% CSE for an additional 24 h. Ten micrometres DCF-DA was added into each well and the plate was incubated for 30 min in dark environment. The fluorescence intensity was calculated using the FLUOstar Omega at excitation wavelength of 488 nm and the emission wavelength of 525 nm (Paudel et al., 2020).

#### Fluorescence imaging

RAW 264.7 cells were cultured using a cover slip on a 6-well plate. The cells were then pre-treated for 1 h with 5 or 10 μM of pure ZER or ZER-LCNs, followed by incubation with 5% CSE for a duration of 24 h. The cells were then washed twice with PBS and left in a dark environment for 30 min with 10 μM DCF-DA. The cells were washed with PBS and fluorescence images were immediately captured at ×20 magnification using fluorescence microscope (Zeiss Axio Imager Z2, Oberkochen, Germany) (Paudel et al., 2020).

### Human cytokines protein array

In a 6-well plate, the BCi-NS1.1 cells were pre-treated with or without various concentration of the pure ZER or ZER-LCNs at 10 μM followed by incubation with 5% CSE for a duration of 24 h. RIPA lysis buffer was used to lyse and extract the total cellular protein in each well. The protein concentrations were then quantified using Pierce™ BCA protein assay kit (catalogue 23225, Thermo Fisher Scientific) according to manufacturer’s protocol. An equal amount of protein was used from each treatment group to produce blots using the R&D Systems Proteome Profiler Human XL Cytokine Array Kit (R&D Systems, Minneapolis, MN, USA), following the directions provided by the manufacturer.

### Nitric oxide (NO) assay

Using the Griess reagent method, the NO production was determined in the RAW 264.7 cells. The cells were seeded in 96-well plate and pre-treated with different concentrations of the pure ZER or ZER-LCNs at 5 or 10 μM, followed by the 24 h exposure to 5% CSE. One hundred microlitres of the culture supernatant media was taken out and added to the 100 μL Griess reagent (1:1 ratio). Using the FLUOstar Omega Reader, the absorbance was measured at 540 nm to obtain the optical density of the colour product. The amount of nitrite in the supernatant media was calculated by comparing the absorbance data against the standard curve of NaNO_3_

### Real time qPCR

In a 6-well plate, the RAW 264.7 cells and BCi-NS1.1 cells were cultured and grown separately. The cells were pre-treated with or without pure ZER or ZER-LCNs at 10 μM for 1 h followed by exposure to 5% CSE for an additional 24 h. The cells were lysed with TRIzol for the isolation of total RNA. Following this, using reverse transcription of RNA (200 ng), the cDNA was synthesised. The cDNA was then processed using the real-time quantitative PCR analysis to identify the gene expression measurement. Then the gene expression was measured using 2^−[∆∆]Ct^ to correlate with the relevant genes (Paudel et al., 2022c).

### X-gal staining

In the glass cover slip of 6-well plates, the BCi-NS1.1 cells were grown. The cells were pre-treated for 1 h with pure ZER or ZER-LCNs at the concentration of 10 μM, which was then followed up by the 24 h exposure to 5% CSE. After that, the cells were washed with PBS followed by the addition of the fixative solution (supplied in the kit, ab102534) at room temperature for a duration of 10 min. Using a staining mixture (staining solution, staining supplement and X-gal), the cells were then stained overnight at 37 °C. This was then followed by transferring the coverslips from the plates onto a glass slide. Images were then captured using a Zeiss Axio Imager Z2 microscope at ×20 magnification

### Immunocytochemistry of p21 and p16

In the glass cover slip of 6-well plate, the BCi-NS1.1 cells were cultured. The cells were pre-treated for 1 h with pure ZER or ZER-LCNs at the concentration of 10 μM, which was then followed up by the 24 h exposure to 5% CSE. After which, the cells were washed with PBS and followed by fixing using 4% paraformaldehyde for 10 min, permeabilised with 0.5% Triton X-100 for 30 min, blocking with 1% bovine serum albumin for another 30 min. Then, using a 1:800 dilution, the cells were incubated with anti-p21 or anti-p16 (Cell Signalling Technology) overnight at 4 °C and followed the next day with goat antirabbit Alexa488 (for p16) and Alexa 647 (for p21) at a dilution of 1:1000 for a duration of 1 h. Then, the cover slips were mounted with fluoro-mount containing 4′,6-Diamidino-2-phenylindole (DAPI) which were used for nuclear staining. This was then followed with images of cells that were taken with Zeiss Axio Imager Z2 microscope (Oberkochen, Germany) at a magnification of ×40, and mean fluorescence intensity was quantified using Image J software

### Statistical analysis

The results were presented as mean ± SEM. The statistical analysis was performed by one-way ANOVA followed by Dunnett’s or Tukey’s multiple comparison test using the Graph Pad Prism software (version 9.3). The *p* < 0.05 was considered statistically significant.

## Results

### Effect of ZER and ZER-LCNs on the viability of CSE-induced RAW264.7 cells

**Fig. 2:**
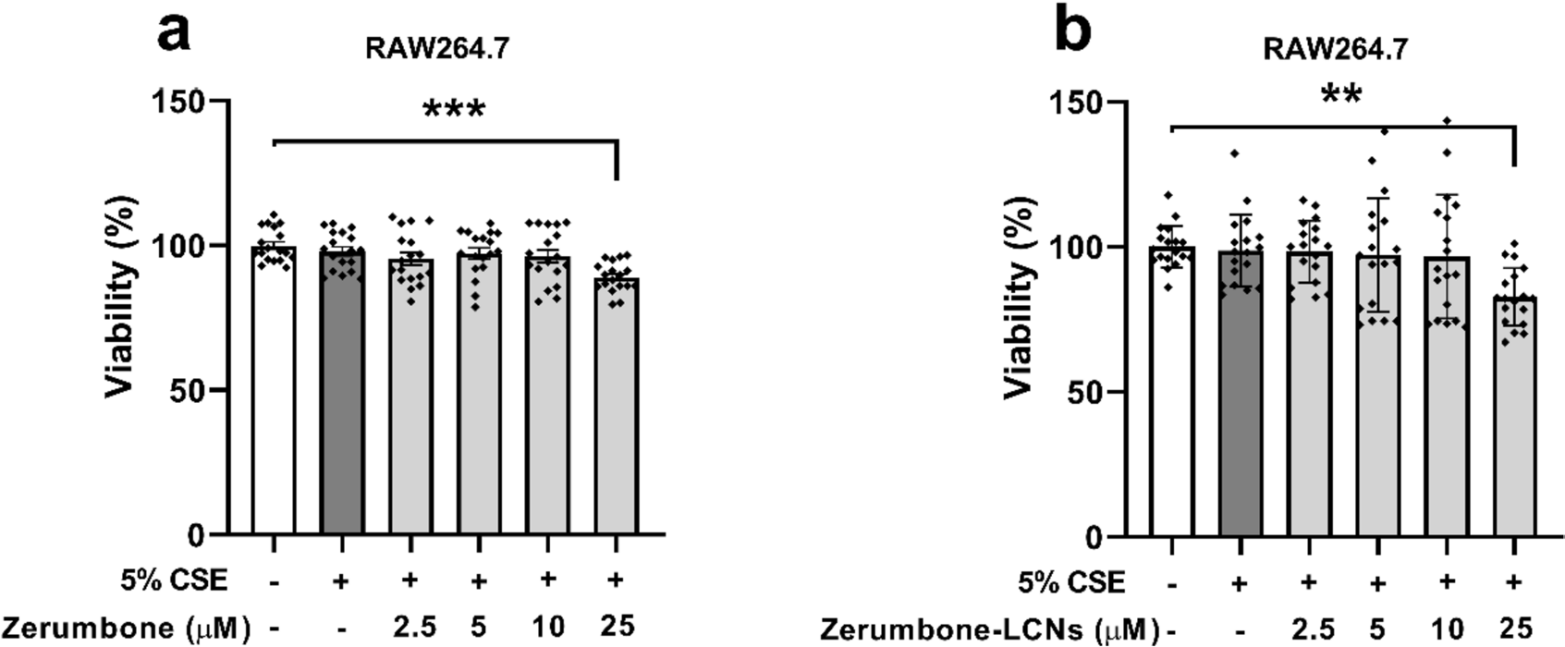
Effect of ZER and ZER-LCNs on viability of RAW264.7 cells. RAW264.7 cells were pre-treated for 1 h with or without ZER (**a**) or ZER-LCNs (**b**) at final concentration of 2.5, 5, 10 or 25 μM, followed by 24 h incubation in the absence or presence of 5% CSE. The cell viability was measured by incubating the cells with MTT and measuring the absorbance of purple formazan at a wavelength of 540 nm with a microplate reader. Values are expressed as mean ± SEM from 3 independent experiments. Statistical analysis was performed with one-way ANOVA followed by Dunnett’s multiple comparison test. ** *p* < 0.01, ***p < 0.001. CSE, cigarette smoke extract; zerumbone-LCNs, zerumbone-liquid crystalline nanoparticles

The toxicities of ZER and ZER-LCNs on 5% CSE-induced RAW264.7 cells up to a concentration of 25 μM were investigated to determine their optimal non-toxic dose for treatment. Both ZER and ZER-LCNs did not show cell toxicity up to a concentration of 10 μM (Fig. 2a, b). However, the cell viability was significantly decreased when 5% CSE-induced RAW264.7 cells were incubated with ZER (Fig. 2a, *p* < 0.001 against control) or ZER-LCNs (Fig. 2b, *p* < 0.01) at a concentration of 25 μM. Hence, both ZER and ZER-LCNs were used at 5 and 10 μM concentrations in the subsequent experiments.

### ZER and ZER-LCNs inhibit CSE-induced ROS generation in RAW264.7 cells

**Fig. 3:**
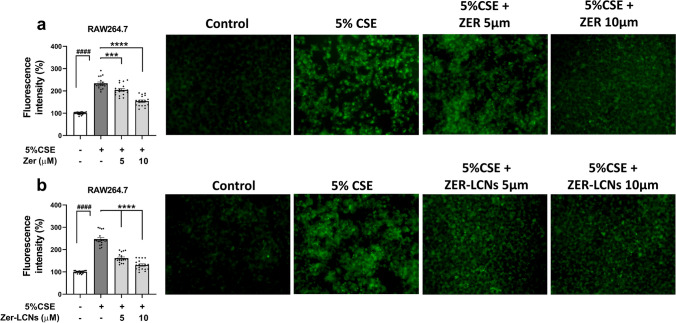
Effect of free ZER and ZER-LCNs on ROS production in RAW264.7 cells. RAW264.7 cells were pre-treated for 1 h with or without ZER (**a**) or ZER-LCNs (**b**) at final concentration of 5 and 10 μM, followed by 24 h incubation in the absence or presence of 5% CSE. The fluorescence intensity of DCF-DA was measured using fluorescence plate reader and the fluorescence images were acquired using fluorescence microscopy at ×20 magnification. Values are expressed as mean ± SEM of 3 independent experiments. Analysis was performed with one-way ANOVA followed by Dunnett’s multiple comparison test. ^####^*p* < 0.0001 vs control (without 5% CSE); ***p < 0.001, ****p < 0.0001 vs 5% CSE (without zerumbone/zerumbone-LCNs). CSE, cigarette smoke extract; zerumbone-LCNs, zerumbone-liquid crystalline nanoparticles

The results from DCF-DA fluorescence suggest that the ROS production was elevated by > 2.4-fold in RAW264.7 cells exposed to 5% CSE for 24 h in comparison to the control group (Fig. 3a, b, *p* < 0.0001 in both). Both free ZER and ZER-LCNs dose dependently decreased the 5% CSE-induced ROS generation (Fig. 3a, b, *p* < 0.0001 for both, at 5 and 10 μM concentrations). However, the effect of ZER-LCNs on decreasing the ROS generation was comparatively higher than that of free ZER (Fig. 3a, b). This is consistently observed in the fluorescence images, where ZER-LCNs significantly lowered the ROS intensity at both 5 and 10 μM concentrations in comparison to free ZER.

### ZER and ZER-LCNs inhibit CSE-induced NO production in RAW264.7 cells

**Fig. 4:**
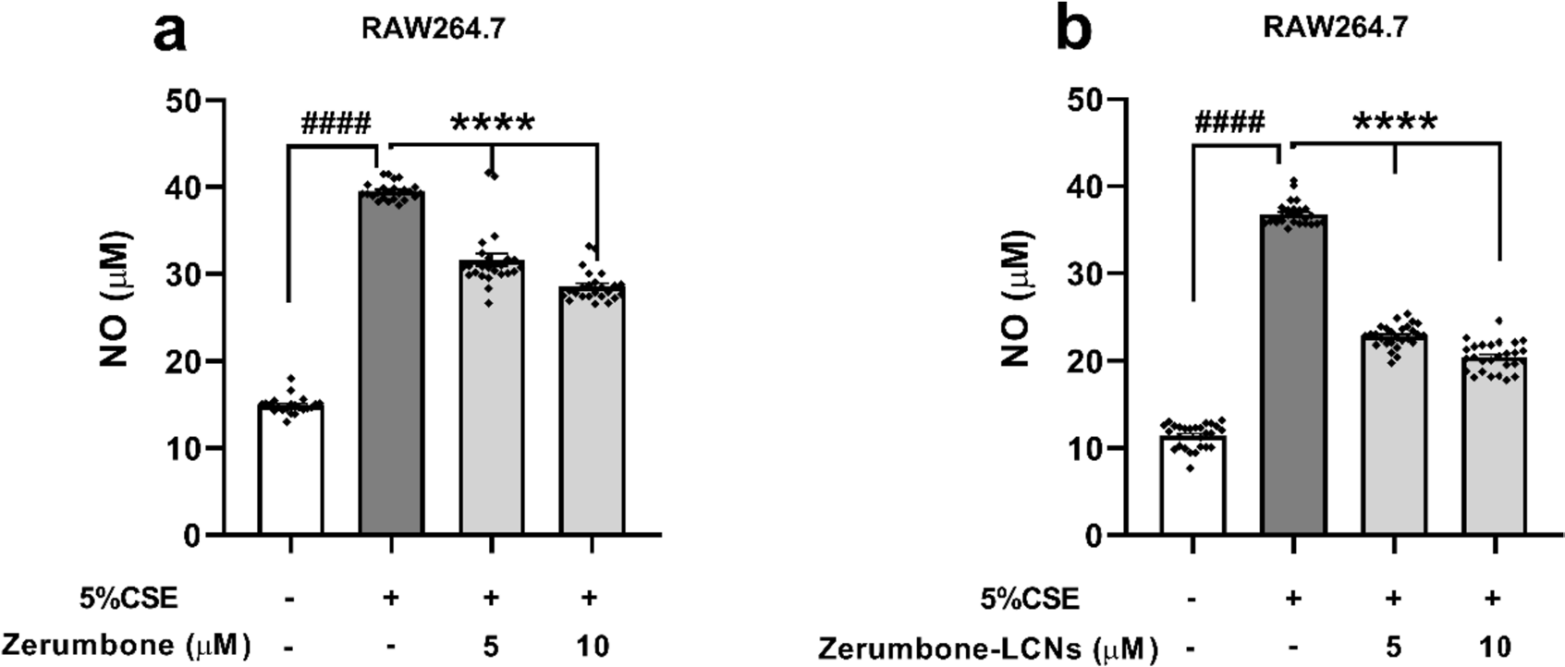
Effect of free ZER and ZER-LCNs on NO production in RAW264.7 cells. RAW264.7 cells were pre-treated for 1 h with or without ZER (**a**) or ZER-LCNs (**b**) at final concentration of 5 and 10 μM, followed by 24 h incubation in the absence or presence of 5% CSE. NO production was quantified in terms of nitrite with Griess’ reagent by measuring absorbance at 540 nm with microplate reader. Values are expressed as mean ± SEM of 3 independent experiments. Analysis was performed with one-way ANOVA followed by Dunnett’s multiple comparison test. ^####^*p* < 0.0001 vs control (without 5% CSE), ****p < 0.0001 vs 5% CSE (without zerumbone/zerumbone-LCNs). CSE, cigarette smoke extract; zerumbone-LCNs, zerumbone-liquid crystalline nanoparticles

The results from NO assay suggested that RAW264.7 cells treated with 5% CSE for a duration of 24 h resulted in an approximate 3-fold increase of NO level in the cells as compared to the control (in the absence of CSE). Both ZER-LCNs formulation and free ZER were able to significantly decrease the level of nitric oxide at both 5 and 10 μM concentrations (Fig. 4a, b). However, it was also observed that the ZER-LCNs were able to significantly reduce the NO production more than the free ZER at both 5 and 10 μM (Fig. 4a, b).

### ZER and ZER-LCNs regulate the expression of genes related to inflammation in CSE-induced RAW264.7 cells

**Fig. 5:**
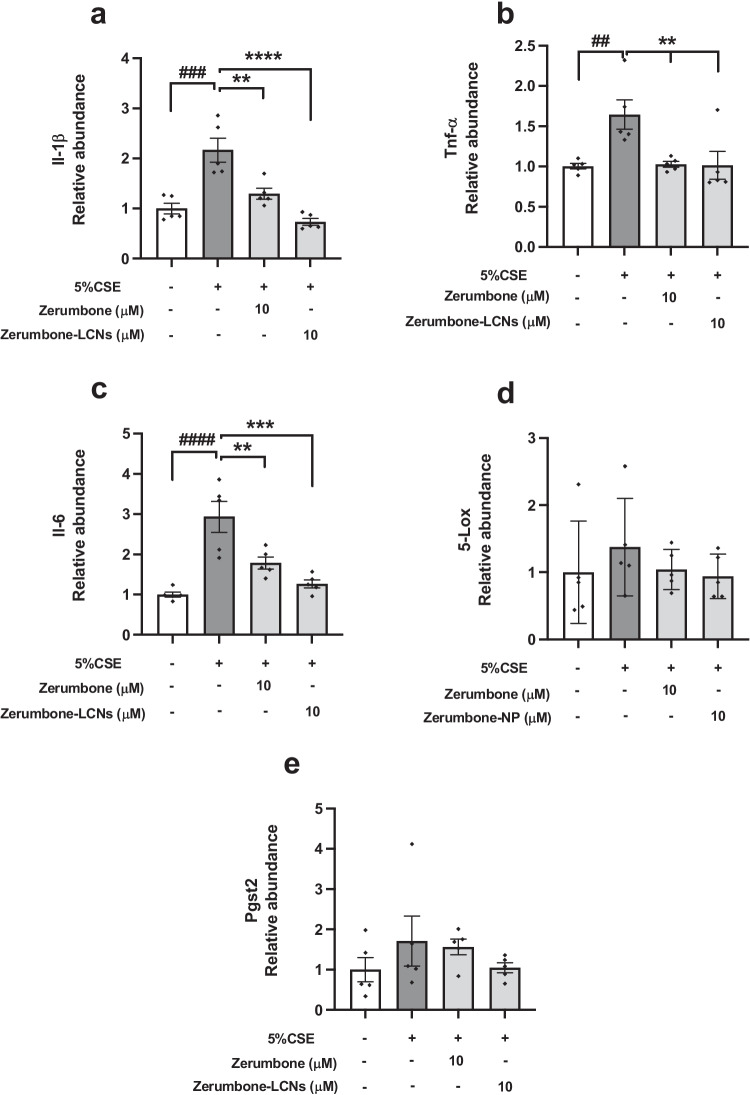
Regulation of expression of genes related to inflammation by ZER and ZER-LCNs in RAW264.7 cells. RAW264.7 cells were pre-treated for 1 h with or without ZER or ZER-LCNs at final concentration of 10 μM, followed by 24 h incubation in the absence or presence of 5% CSE. The mRNA levels of genes encoding IL-1β (**a**), *Tnf-α* (**b**), *Il-6* (**c**), *Psgt2* (**d**) and *5-Lox* (**e**) were determined with RT-qPCR. Values are expressed as mean ± SEM of 3 independent experiments. Analysis was performed with one-way ANOVA followed by Dunnett’s multiple comparison test. ^##^*p* < 0.01, ^###^*p* < 0.001, ^####^*p* < 0.0001 vs control (without 5% CSE); **p < 0.01, ***p < 0.001, ****p < 0.0001 vs 5% CSE (without zerumbone/zerumbone-LCNs). CSE, cigarette smoke extract; zerumbone-LCNs, zerumbone-liquid crystalline nanoparticles

Some of the main pro-inflammatory cytokines involved in COPD inflammation due to cigarette smoking include IL-1β, IL-6 and TNF-α (Paudel et al., 2022c). The results from this study indicated that the RAW264.7 cells that were exposed to 5% CSE had a substantial increase in mRNA levels of these pro-inflammatory cytokines (Fig. 5a, e). The mRNA levels of *Il-1β*, *Tnf-α* and *Il-6* in 5% CSE-induced RAW264.7 cells were increased by 2-fold (Fig. 5a, *p* < 0.001), 1.7-fold (Fig. 5b, *p* < 0.01) and 2.9-fold (Fig. 5c, *p* < 0.0001) in comparison to the untreated control group. With the treatment of free ZER and ZER-LCNs at 10 μM, there was a significant decrease in the mRNA levels of *Il-1β* (Fig 5a), *Tnf-α* (Fig. 5b) and *Il-6* (Fig. 5c). However, RAW264.7 cells incubated with ZER-LCNs showed a greater decrease in mRNA levels of *Il-1β* (Fig. 5a) and *Il-6* (Fig. 5c). For *Tnf-α*, both the free ZER and ZER-LCNs were able to reduce the mRNA levels in a similar manner when treated at a concentration of 10 μM (Fig. 5b).

It was also observed that there was an increase in the PSGT2 which is a product of the cyclooxygenase (COX)-2. PSGT2 causes greater airflow limitations in COPD patients. On the other hand, the 5-lipoxygenase (5-LOX) also contributes to inflammation of COPD. The results in this study suggested that *Psgt2* mRNA levels were not affected by 5% CSE (Fig. 5d). However, mRNA levels of *5-Lox* were significantly upregulated in RAW264.7 cells exposed to 5% CSE as compared to the control (Fig. 5e). There was no effect of ZER and ZER-LCNs on the regulation of *Psgt2* and *5-Lox* (Fig. 5d, e).

### ZER and ZER-LCNs regulate the expression of antioxidant genes in CSE-induced RAW264.7 cells

**Fig. 6:**
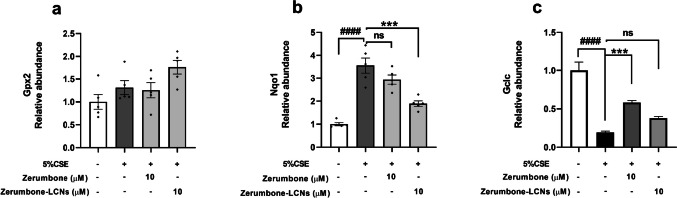
Regulation of gene expression related to oxidative-stress by ZER and ZER-LCNs. RAW264.7 cells were pre-treated for 1 h with or without ZER or ZER-LCNs at final concentration of 10 μM, followed by 24 h incubation in the absence or presence of 5% CSE. The mRNA levels of genes encoding *Gpx2* (**a**), *Nqo1* (**b**) and *Gclc* (**c**) were determined with RT-qPCR. Values are expressed as mean ± SEM of 3 independent experiments. Analysis was performed with one-way ANOVA followed by Dunnett’s multiple comparison test. ^####^*p* < 0.0001 vs control (without 5% CSE), ***p < 0.001 vs 5% CSE (without zerumbone/zerumbone-LCNs). CSE, cigarette smoke extract; zerumbone-LCNs, zerumbone-liquid crystalline nanoparticles

The mRNA levels of antioxidants such as *Gpx2*, *Nqo1* and *Gclc* and their regulation by ZER and ZER-LCNs were also investigated in this study. Gpx2 is a common antioxidant enzyme, and it is commonly found to increase during oxidative stress as a compensatory mechanism against the toxic oxidants that are generated by cigarette smoke. The results indicated that the 5% cigarette smoke increased *Gpx2* in RAW264.7 cells; however, both the free ZER and ZER-LCNs at 10 μM were decrease *Gpx2* mRNA expression (Fig. 6a). There was an approximately 3.5-fold increase in the *Nqo1* mRNA expression in RAW264.7 cells treated with 5% CSE (Fig. 6b). Ten micrometres free ZER and ZER-LCNs, respectively, it was observed that there was a decrease in the levels of *Nqo1*, with a much greater decrease observed for ZER-LCNs as observed in Fig. 6b. Lastly, the GCLC factor was also investigated, whereby when the cells were exposed to 5% CSE, the levels of *Gclc* significantly reduced. As observed in Fig. 6c, when the cells were then treated with 10 μM free ZER and ZER-LCNs, the free ZER was able to increase the amount of *Gclc* greater than the ZER-LCNs.

### Effect of ZER and ZER-LCNs on the viability of CSE-induced BCi-NS1.1 cells

**Fig. 7:**
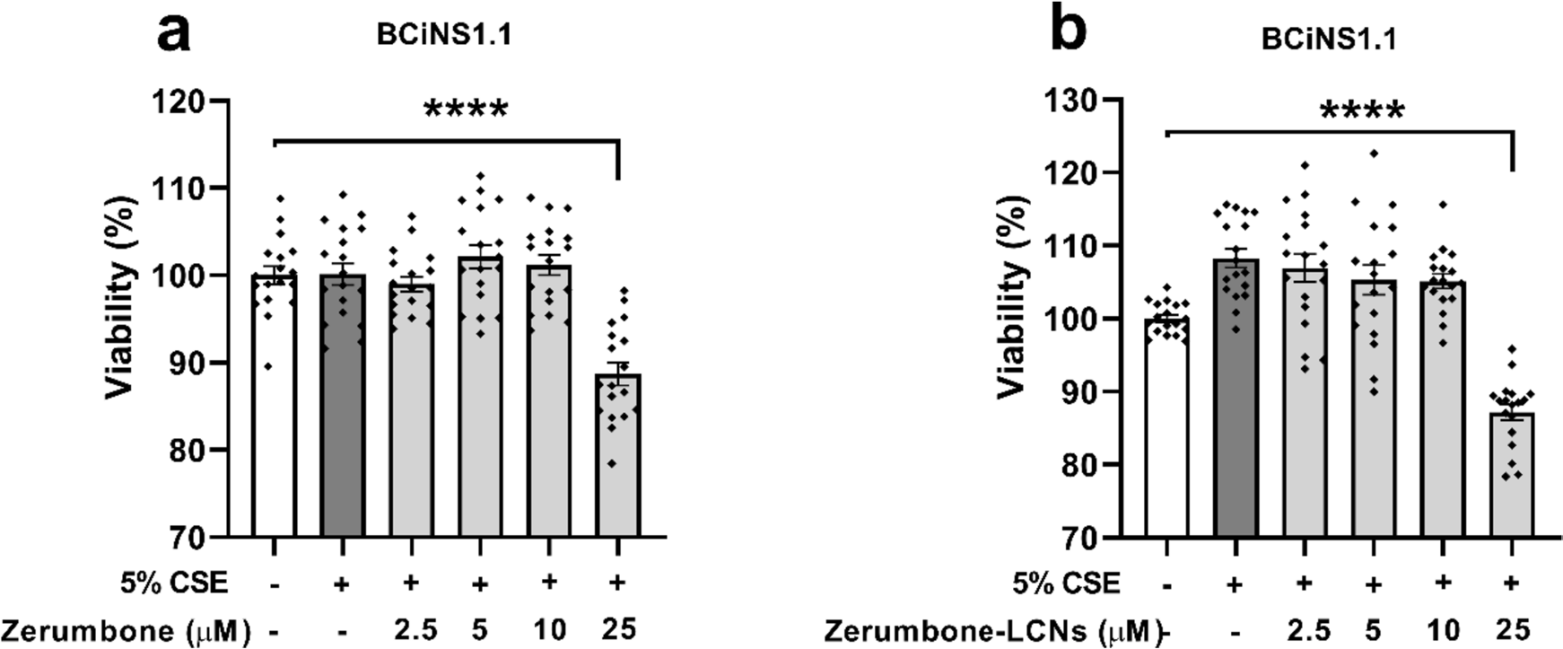
Effect of ZER and ZER-LCNs on the viability of BCi-NS1.1 cells. BCi-NS1.1 cells were pre-treated for 1 h with or without ZER (**a**) or ZER-LCNs (**b**) at final concentration of 2.5, 5, 10 or 25 μM, followed by 24 h incubation in the absence or presence of 5% CSE. The cell viability was measured by incubating the cells with MTT and measuring the absorbance of purple formazan at a wavelength of 540 nm with a microplate reader. Values are expressed as mean ± SEM from 3 independent experiments. Statistical analysis was performed with one-way ANOVA followed by Dunnett’s multiple comparison test. ****p < 0.0001 vs control (without zerumbone/zerumbone-LCNs and 5% CSE treated). CSE, cigarette smoke extract; zerumbone-LCNs, zerumbone-liquid crystalline nanoparticles

Various doses of ZER and ZER-LCNs were tested for the cell viability study on BCi-NS1.1 cell line. Both ZER and ZER-LCNs showed no cell toxicity up to a concentration of 10 μM (Fig. 7). However, at a concentration of 25 μM of ZER or ZER-LCNs, BCi-NS1.1 showed a significant reduction in cell viability (Fig. 7). Hence, the subsequent studies were conducted with ZER or ZER-LCNs at concentration of 10 μM.

### ZER and ZER-LCNs regulates the expression of genes related to inflammation in CSE-induced BCi-NS1.1 cells

**Fig. 8:**
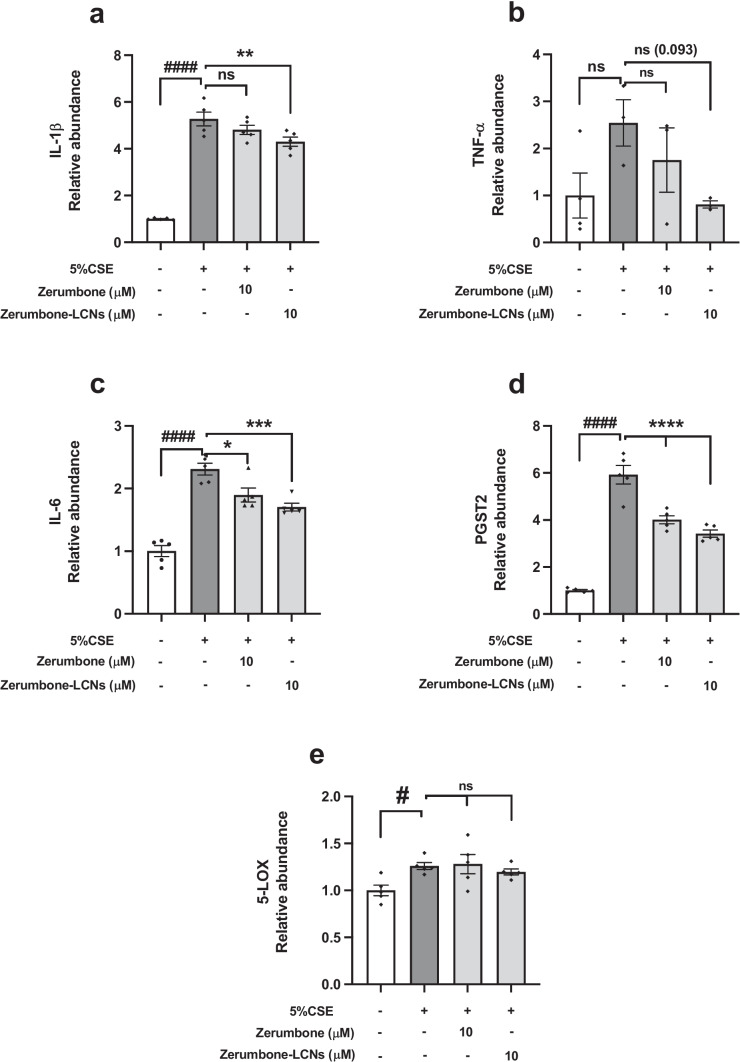
Regulation of expression of genes related to inflammation by ZER and ZER-LCNs in BCi-NS1.1 cells. BCi-NS1.1 cells were pre-treated for 1 h with or without ZER or ZER-LCNs at final concentration of 10 μM, followed by 24 h incubation in the absence or presence of 5% CSE. The mRNA levels of genes encoding *IL-1β* (**a**), *TNF-α* (**b**), *IL-6* (**c**), *PSGT2* (**d**) and *5-LOX* (**e**) were determined with RT-qPCR. Values are expressed as mean ± SEM of 3 independent experiments. Analysis was performed with one-way ANOVA followed by Dunnett’s multiple comparison test. ^####^*p* < 0.0001 vs control (without 5% CSE), *p < 0.05, **p < 0.01, ***p < 0.001, ****p < 0.0001 vs 5% CSE (without zerumbone/zerumbone-LCNs). CSE, cigarette smoke extract; zerumbone-LCNs, zerumbone-liquid crystalline nanoparticles

The gene expression of main inflammatory cytokines *IL-1β*, *IL-6* and *TNF-α* was studied. After exposure to 5% CSE, the mRNA levels of *IL-1β* and *IL-*6 were found to be significantly increased by 5-fold (Fig. 8a, *p* < 0.0001) and 2.2-fold (Fig. 8c, *p* < 0.0001) in the BCi-NS1.1 cells. However, there was no change in *TNF-α* gene expression following 5% CSE or the with the addition of ZER or ZER-LCNs (Fig. 8b). While the gene expression of IL-1β was only reduced by the treatment of ZER-LCNs (Fig 8a), *IL-6* was reduced by the addition of both ZER and ZER-LCNs (Fig. 8c). In addition, the effects of ZER and ZER-LCNs on *PGST2* and *5-LOX* in the BCi-NS1.1 cells were studied as both of these mediators significantly contribute to inflammation. For PGST2, the 5% CSE increased the production of *PGST2* by almost 6-fold (Fig. 8d), and once treated with ZER and ZER-LCNs, it significantly decreased with the greater effect from ZER-LCNs. While *5-LOX* was significantly increased by 5% CSE (Fig. 8e), there was no significant effect on the *5-LOX* genes when treated with both ZER and ZER-LCNs.

### ZER and ZER-LCNs regulates the expression of antioxidant genes in CSE-induced BCi-NS1.1 cells

**Fig. 9:**
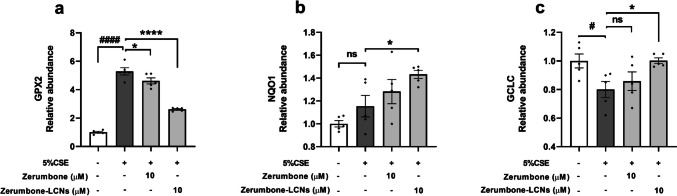
Regulation of gene expression related to oxidative-stress by ZER and ZER-LCNs in BCi-NS 1.1 cells. BCi-NS1.1 cells were pre-treated for 1 h with or without ZER or ZER-LCNs at final concentration of 10 μM, followed by 24 h incubation in the absence or presence of 5% CSE. The mRNA levels of genes encoding *GPX2* (**a**), *NQO1* (**b**) and *GCLC* (**c**) were determined with RT-qPCR. Values are expressed as mean ± SEM of 3 independent experiments. Analysis was performed with one-way ANOVA followed by Dunnett’s multiple comparison test. ^#^*p* < 0.05, ^####^*p* < 0.0001 vs control (without 5% CSE), *p < 0.05, ****p < 0.0001 vs 5% CSE (without zerumbone/zerumbone-LCNs). CSE, Cigarette smoke extract; zerumbone-LCNs, zerumbone-liquid crystalline nanoparticles

The BCi-NS1.1 cells were also tested for the same antioxidant genes as highlighted in RAW264.7 cells (Fig. 6a, c). There was a 5-fold in GPX2 when 5% CSE was introduced to the BCi-NS1.1 cells (Fig. 9a). The treatment with ZER only slightly decreased the GPX2; however, ZER-LCN was able to substantially decrease the amount of GPX2 gene expression (Fig. 9a). The NQO1 gene was not significantly increased when exposed to 5% CSE, and both the ZER and the ZER-LCNs were not able to decrease the expression of this gene (Fig. 9b). Lastly, when 5% CSE was exposed to the BCi-NS1.1 cells, the GCLC gene expression was decreased (Fig. 9c). ZER was able to slightly increase the production of this gene, and ZER-LCN was able to bring back the GCLC level back to its original amount (Fig. 9c).

### ZER and ZER-LCNs regulates the CSE-induced senescence in BCi-NS1.1 cells

**Fig. 10:**
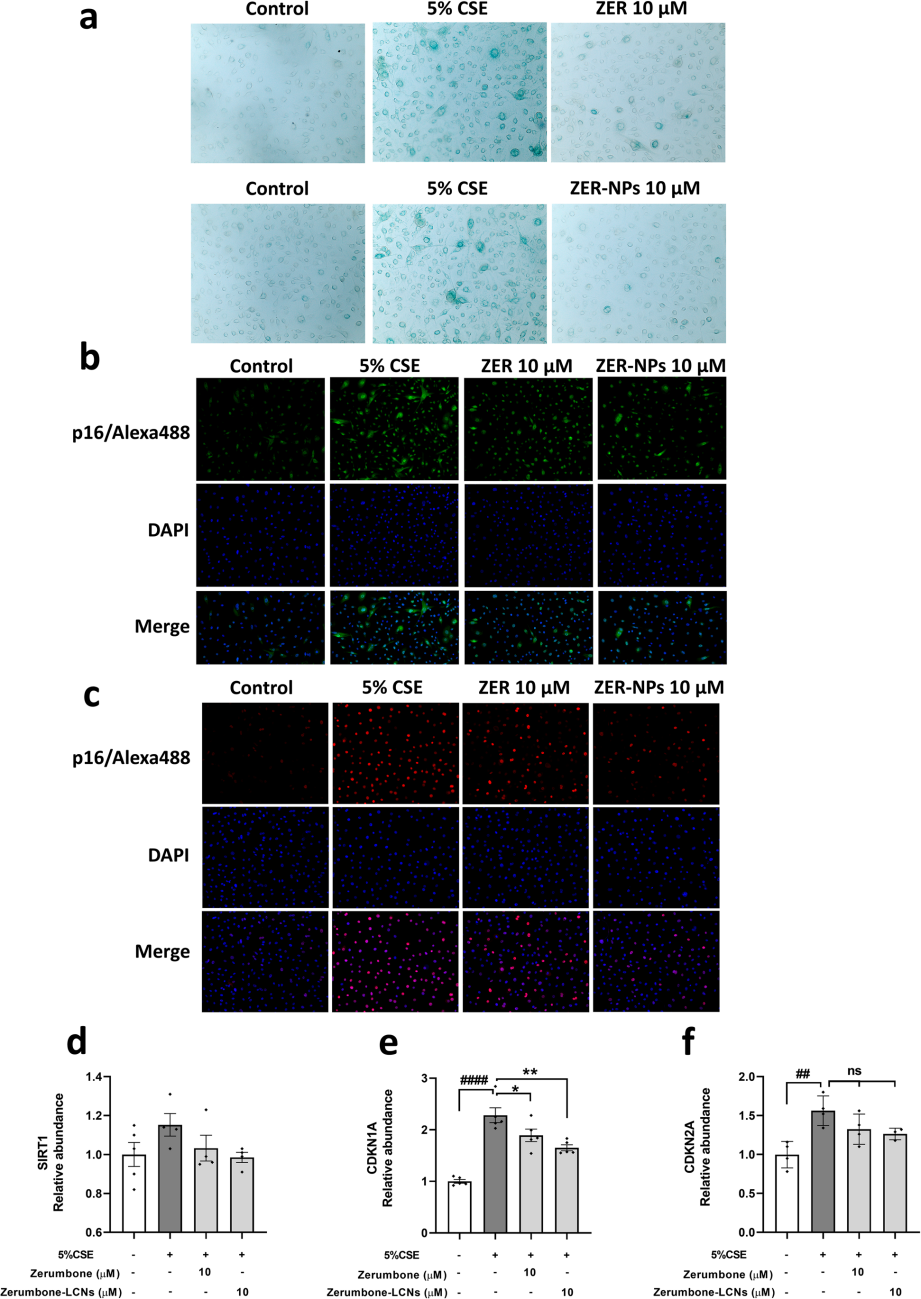
The effect of ZER and ZER-LCNs on CSE-induced senescence on BCi-NS1.1 cells. BCi-NS1.1 cells were pre-treated for 1 h with or without ZER or ZER-LCNs at final concentration of 10 μM, followed by 24 h incubation in the absence or presence of 5% CSE. The microscopic image was taken with Zeiss Axio Imager Z2 microscope at ×20 magnification. The representative images of X-gal staining. The X-gal positive senescence cells are showing with black arrow (**a**), immunofluorescence staining of p16/Alexa488 (**b**) and immunofluorescence staining of p21/Alexa647 (**c**) are shown. The relative gene expression of *SIRT1* (**d**), *CDKN1A* (**e**) and *CDKN2A* (**f**) are shown. Values in **d**–**f** are expressed as mean ± SEM of 3 independent experiments. Analysis was performed with one-way ANOVA followed by Dunnett’s multiple comparison test. ^##^*p* < 0.01, ^####^*p* < 0.0001 vs control (without 5% CSE), *p < 0.05, **p < 0.01 vs 5% CSE (without zerumbone/zerumbone-LCNs). CSE, cigarette smoke extract; zerumbone-LCNs, zerumbone-liquid crystalline nanoparticles.

The effects of ZER and ZER-LCNs to halt the senescence induced by 5% CSE was also tested on BCiNS1.1 cells using senescence associated X-gal staining (Fig. 10a), immunofluorescence staining of p16 (Fig. 10b), p21 (Fig. 10c) and genes expression of anti-aging *SIRT1* gene (Fig. 10d), and senescence markers *CDKN1A* (p21) (Fig. 10e) and the *CDKN2A* (p16) (Fig. 10f). The exposure of 5% CSE to the BCiNS1.1 cells resulted in the X-gal positive blue staining of senescent cells while treatment of pure ZER and ZER-LCNs at 10 μM decreased the number of X-gal positive cells (Fig. 10a). It is interesting to note that the size of senescence positive cells is larger than other cells, a common feature of senescence where cell remain viable and slightly bigger in size. Similarly, the both protein (fluorescence staining) and gene expression of p16 and p21 was upregulated by 5% CSE while, ZER and ZER-LCNs significantly decreased the expression of p16 (Fig. 10b, f) and p21 (Fig. 10c, e). Comparatively, the effect of ZER-LCNs was superior to the pure ZER. However, there was no changes in the expression of SIRT gene expression by 5% CSE, ZER and ZER-LCNs (Fig. 10d).

### ZER and ZER-LCNs regulates the expression of proteins related to CSE-induced inflammation in BCi-NS1.1 cells

**Fig. 11:**
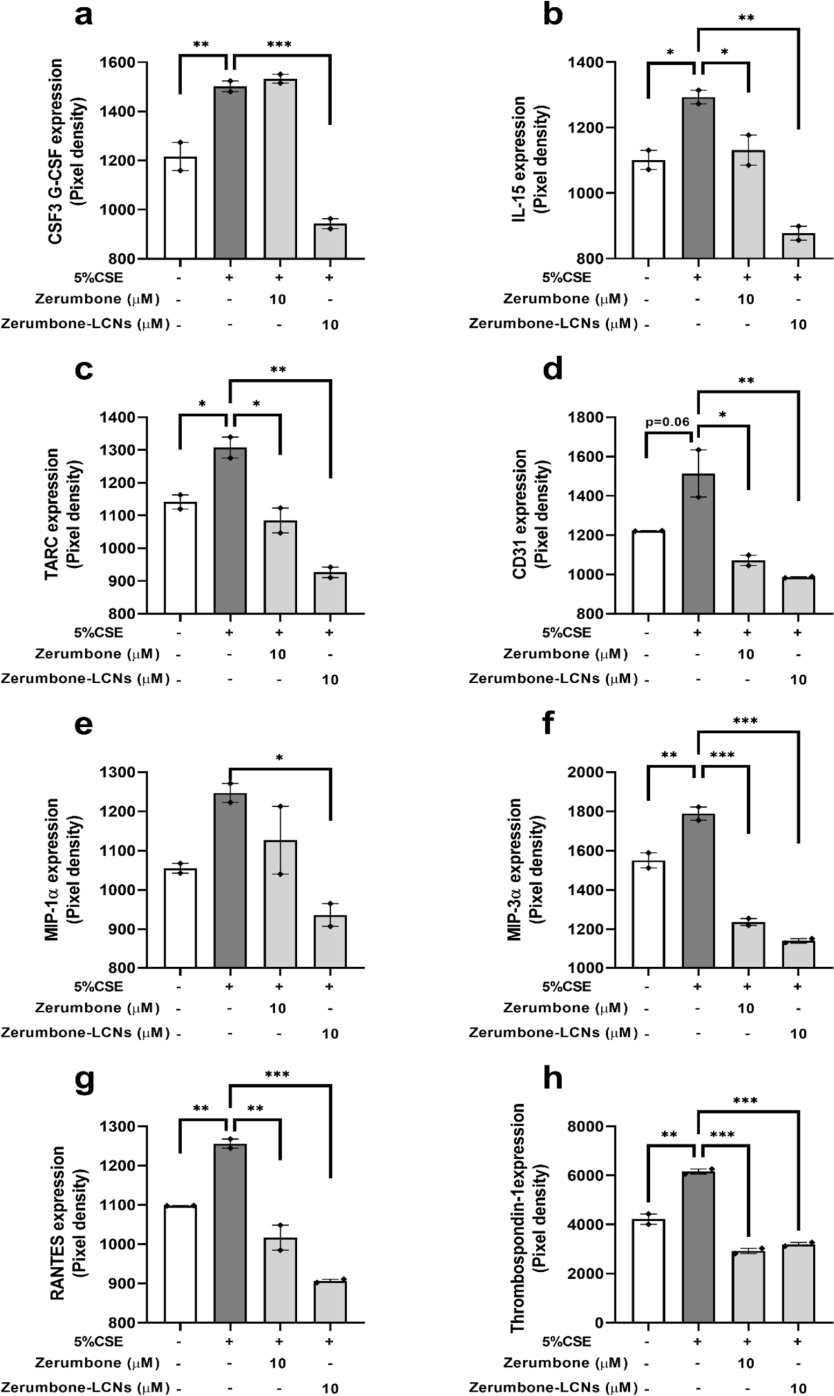
Effect of ZER and ZER-LCNs on the expression of proteins related to inflammation in BCi-NS1.1 cells. BCi-NS1.1 cells were pre-treated for 1 h with or without ZER or ZER-LCNs at final concentration of 10 μM, followed by 24 h incubation in the absence or presence of 5% CSE. The cytokine protein array kit was used to identify the proteins regulated by 5% CSE and ZER and ZER-LCNs. The relative protein expression of CSF3 (**a**), IL-15 (**b**), TARC (**c**), CD31 (**d**), MIP-1α (**e**), MIP-3α (**f**), RANTES (**g**) and TSP-1 (**h**) upon the treatment with zerumbone and ZER-LCNs on BCi-NS1.1 cells is shown. Values are expressed as mean ± SEM of 2 independent experiments. Analysis was performed with one-way ANOVA followed by Dunnett’s multiple comparison test. *p < 0.05, **p < 0.01, ***p <0.001. CSE, cigarette smoke extract; zerumbone-LCNs: zerumbone-liquid crystalline nanoparticles

The cytokines protein array kit was used to identify the proteins that may play a role in the inflammatory response of COPD in BCi-NS1.1 cells. The cells subjected to 5% CSE showed a significant increase in granulocyte-CSF CSF3 (Fig. 11a) , IL-15 (Fig. 11b), thymus and activation-regulated chemokine (TARC, Fig. 11c), macrophage inflammatory protein (MIP)-3α (Fig. 11f), RANTES (Fig. 11g) and thrombospondin-1 (TSP-1, Fig. 11h). A trend of increase in protein expression of cluster of differentiation 31 (CD31, Fig. 11d) and MIP-1α (Fig. 11e) was observed in 5% CSE-treated cells. The introduction of ZER did not decrease the CSF3 or MIP-1α expressions; however, ZER-LCNs significantly reduced expressions of both of these proteins (Fig. 11a, e). The protein expression of IL-15, TARC, CD31, MIP-3α, regulated on activation, normal T cell expressed and secreted (RANTES) and TSP-1 was significantly decreased by both ZER and ZER-LCN with superior effect shown by ZER-LCNs (Fig. 11b, d and f, h). The expression of TSP-1, however, was reduced by both ZER and ZER-LCN in equal levels (Fig. 11h).

## Discussion

One of the main aims of our research is to investigate the medicinal benefits of ZER-LCNs against COPD by utilising CSE-induced *in vitro* models of RAW264.7 and BCi-NS1.1 cells. The finding of this study suggested that ZER-LCNs exhibit anti-inflammatory, antioxidant and anti-senescence activities against the cigarette-smoke-induced inflammation and oxidative stress in RAW264.7 and BCi-NS1.1 cells. The gene and protein expressions related to COPD were investigated to identify the mechanisms underlying the protective activity of the ZER-LCNs in these cells.

CSE induces inflammatory responses in the lungs potentially leading to lung diseases (Mallampalli et al., 2020). The inflammation causes increase in the number of macrophages in the airways as well as the alveolar septa and cavities (Bao et al., 2020). Previous studies have indicated that airway inflammation can lead to the release of pro-inflammatory cytokines such as IL-1β, IL-6 and TNF-α by the human broncho-epithelial cells and macrophages (Tamimi et al., 2012). In our current study, the strong anti-inflammatory activity of ZER-LCN was caused by the inhibition of cytokines and other mediators in both BCi-NS1.1 cells as well as the RAW264.7 cells lines. The RAW 264.7 cells are rooted from Abelson leukaemia virus transformed cell lines which are derived from BALB/c mice. They show the appropriate characteristics to be used in this study as they can perform pinocytosis and phagocytosis thus mimicking monocytes and macrophage-like cells (Taciak et al., 2018). Additionally, the BCi-NS1.1 cell line is rooted from the airway epithelium human airway basal cells which is a multipotent progenitor population. These cell lines mimic the normal lung cells such that they are only able to divide for a limited number of times before they move into a state of replicative senescence (Walters et al., 2013).

In BCi-NS1.1 cells, the RT-qPCR analysis showed that ZER-LCNs exhibit very beneficial anti-inflammatory activity due to its ability to inhibit the gene expression of *IL-1β* and *IL-6* and in most cases even more substantially than ZER on its own. Treatment with 5% CSE increased the mRNA levels of PGST2, but when treated with ZER-LCN, it resulted in the decrease of the *PGST2*. PGST2 is a crucial bioactive lipid that is produced by the COX-2 enzyme, and it has a wide range of pharmacological effects on inflammation and cancer thus it can be used as a potential anti-inflammatory target (Nakanishi and Rosenberg, 2013). COX-2 is a common rate-limiting enzyme that is found in the prostanoid pathway which has been considered a key component in the airway inflammation of COPD (Rumzhum and Ammit, 2016). The 5-LOX marker, which is relevant in the pulmonary inflammation, also contributes to the production of proinflammatory leukotrienes through the metabolism of arachidonic acid (Kilfeather, 2002). However, our study did not show any changes in the amount of *5-LOX* when it was treated with ZER-LCN. Adding on to that, NO is a key signalling molecule in the pathogenesis of inflammation in the body and its overproduction is considered as pro-inflammatory mediator. Therefore, an inhibition of NO is crucial for therapeutic management of diseases related to inflammation (Sharma et al., 2007). Our results for ZER-LCN on RAW264.7 cells showed a notable reduction of NO production in the cell, with more exemplary results compared to ZER on its own.

In addition to that, oxidative stress occurs when the amount of ROS in the body exceeds the usual antioxidant defences. The ROS production is significantly increased in lung illnesses usually because of respiratory burst of neutrophils, phagocytes or additional endothelial cells throughout the process of inflammation (de Carvalho et al., 2016). The results from our study showed the antioxidant ability of ZER-LCN in decreasing the total ROS production in the RAW264.7 cells. The ROS overproduction induced by CSE is overcome by the cell defence mechanism that involves the action of GCLC, GPX-2, HO-1 and NQO1. GCLC catalyses the production of glutathione (GSH), which is then converted to GSSG by the catalytic action of GPX-2, resulting in the neutralisation of ROS (Birben et al., 2012). In addition, NQO1 and HO-1 also provide protection against ROS and ROS-mediated oxidative harm (Preethi et al., 2022). It has been observed that the exposure of CSE to cells can substantially upregulate the expression of gene encoding *GPX-2* as a compensatory mechanism for protection against the cigarette smoke oxidant (Bazzini et al., 2013). Our study showed an increase in the expression of gene encoding *GPX2* when exposed to 5% CSE; however, no significant reduction was observed in its expression with treatment of ZER-LCNs. The gene encoding NQO1 is another common gene in both mice and humans that get upregulated in lung tissues when exposed to cigarette smoke (Obeidat et al., 2018). In a study conducted on the bronchial epithelial cells, the *NQO1* gene was upregulated by 5.73-fold when exposed to 5% CSE for a duration of 18 h (Pickett et al., 2010). Similarly, our study showed an upregulation of the *Nqo1* gene by almost 3.5-fold when the RAW 264.7 cells were exposed to 5% CSE for 24 h. When these cells were treated with ZER-LCN, it was observed that there was a notable decrease in the expression of the NQO1 gene which was greater than treatment of free ZER. Besides the *NQO1* gene, the *GCLC* gene which is another important antioxidant gene was also tested. The gene encoding *GCLC* was found to be downregulated in the presence of 5% CSE and subsequently increased when treated with ZER-LCNs.

Similar tests were conducted on the BCi-NS1.1 cell lines, whereby it was observed that there was an upregulation of the gene expression of inflammatory cytokines *IL-1β*, *IL-6* and *TNF-*α when subjected to 5% CSE. These were significantly reduced with ZER-LCN, and at even greater extent than ZER alone treatment. Gene expressions of other inflammatory mediators such as *PGST2* and *5-LOX* were also tested for the same reasoning as in RAW264.7 cells. There was an upregulation for the *PGST2* gene when exposed to 5% CSE but then decreased when treated with ZER-LCNs. There were no notable positive results for *5-LOX* as its gene expression did not reduce when treated with ZER-LCN. A protein array for the inflammatory cytokines was also conducted where several other triggers for the pathogenesis of COPD were identified. Firstly, the G-CSF or CSF3 which has been linked to COPD as without the presence of G-CSF is a notable decrease in airway inflammation as well as lung tissue destruction and many other benefits (Tsantikos et al., 2018). Additionally, the IL-15 cytokine plays a crucial role in the COPD inflammation as it amplifies the type 1 immune response in the respiratory epithelial cells (Zdrenghea et al., 2012). TARC also acts as a biomarker for predicting the decline in pulmonary function (Machida et al., 2021). The CD31 or also known as the platelet endothelial cell adhesion molecule (PECAM-1) plays an vital role during the endothelium repair process in COPD patients (Kato et al., 2014) whereas the chemokines such as MIP-1α, MIP-3α and RANTES are usually found to be upregulated in patients with COPD, thus advocating the idea that they contribute to the pathogenesis of this disease (Bracke et al., 2007). Lastly, TSP-1 is an important mediator for the pathogenesis of COPD. The expression of TSP-1 in individuals with smoking history has been associated with the obstruction and the impairment of the ventilatory pathway (Ishikawa et al., 2013). In our study, the exposure of the BCi-NS1.1 cells to 5% CSE significantly increased several of these inflammation-related protein markers that have been mentioned; however, once treated with ZER-LCNs, it was observed that these mediators were significantly decreased. In fact, in most cases, except for TSP-1, ZER-LCN was able to reduce the expression of these proteins more effectively than the pure ZER.

In terms of the anti-oxidative activity on the BCi-NS1.1 cells, the gene expression was studied on the *GPX2*, *GCLC* and *NQO1*. The cells exposed to 5% CSE showed an upregulation of the *GPX2* and *NQO1* gene expression; however, ZER-LCN was only able to reduce the gene expression in *GPX2* but substantially more than ZER on its own. When tested for *GCLC* gene expression, the 5% CSE reduced the amount of the gene expression, but the ZER-LCN was able to bring it back to its optimal pre-CSE exposure gene levels.

**Fig. 12:**
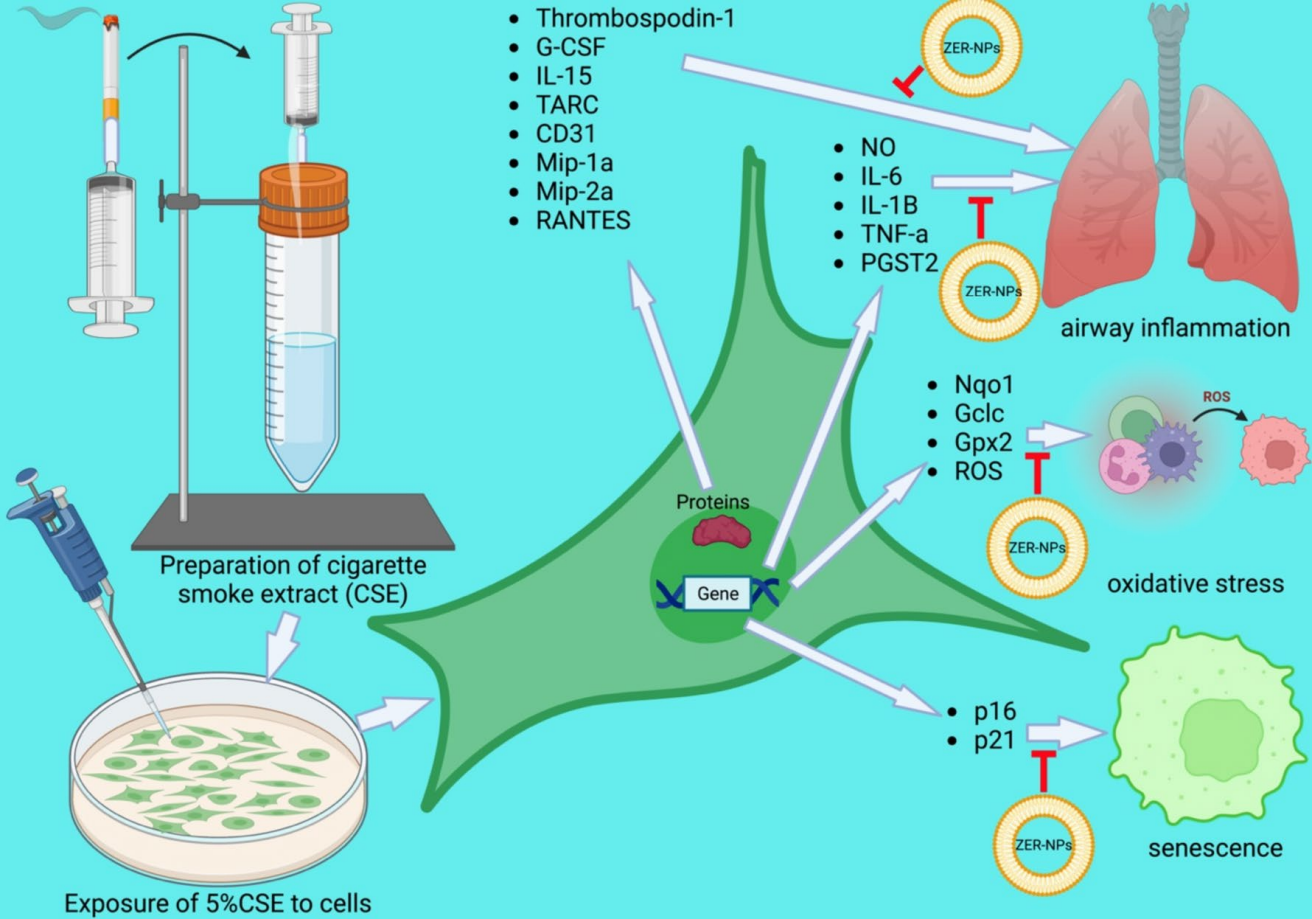
Biological activity of ZER-LCNs *in vitro* in cigarette smoke induced BCi-NS1.1 and RAW264.7 cells

To identify the effects of ZER and ZER-LCN on senescence, an additional study that was conducted on the BCi-NS1.1 cell by the X-gal staining, immunofluorescence staining for p16 and p21 as well as measuring the senescence markers. The antisenescence activity of a potential drug can be tested by observing its markers p21 and p16 (Liu et al., 2016) expression as well as targeting antiaging molecules including SIRT (Grabowska et al., 2017). The SIRT plays a crucial role in protecting the cell against oxidative stress, maintaining and promoting DNA stability and regulating glucose and lipid metabolism thus having association with age-related pathologies (Kilic et al., 2015). Senescence is a multi-step process which requires many mediators, among which two of the key factors include p21 and p16. The upregulation of these factors contributes to the terminal stages of growth arrest in the senescence activity (Alcorta et al., 1996). For the X-gal staining, a positive blue result was observed when the BCi-NS1.1 cells were exposed to 5% CSE which was then substantially reduced when treated with ZER and ZER-LCNs. Following that, the fluorescence staining which was indicative of the protein p16 and p21 was upregulated when exposed to 5% CSE but then significantly reduced when treated with ZER and ZER-LCNs. Lastly, it was found that when exposed to 5% CSE, the *CDKN1A* (p21) and *CDKN2A* (p16) gene expression were increased. However, when treated with ZER-LCN, all three gene expressions were decreased. The gene expression results showed no difference in the *SIRT* expression when subjected to 5% CSE, ZER or even ZER-LCNs. It is also important to note that most of the results showed that ZER-LCNs had greater and more superior effects compared to ZER on its own. The biological activity of ZER-LCNs *in vitro* in cigarette smoke induced BCi-NS1.1 and RAW264.7 is shown in Fig. 12.

The superior biological activities of ZER-LCN observed in this study suggest that it can overcome many of the unfavourable characteristics of pure ZER using the nanoformulation technology. Overall, formulating ZER into LCNs exhibited greater pharmacological and biological benefits in comparison to free ZER on its own in decreasing inflammation, oxidative stress and senescence caused by cigarette smoking. The underlying mechanisms for these effects of ZER-LCNs were shown to involve the transcriptional regulation and protein expression of several key genes including pro-inflammatory cytokines, anti-oxidant enzymes and several markers of inflammation and senescence. However, our study has its own limitations; for example, our study was mainly based on *in vitro* analysis on cell lines. Additionally, there are also various other lung-based cell lines that can be studied for the therapeutic potential of ZER-LCN on COPD such as goblet cells, fibroblast and even the tracheal smooth muscle cells. Moving forward, it would be useful and compelling to conduct *in vivo* biological analysis of ZER in the LCN formulation through the inhalation delivery method in experimental mice model with COPD that have been instigated by cigarette smoke. Using an animal model would also allow us to determine the long-term safety, pharmacokinetics, herb-drug complexities and many other factors as these plays a pivotal part in determining toxicity and the bioavailability of drugs. We should also consider some limitations associated with the CSE study. Firstly, the CSE preparation should be uniform each time for reproducibility. For this, researchers measure the optical density of prepared CSE to get uniform value referring to the similar composition of CSE each time. Secondly, CSE should be prepared fresh each time and it is recommended to expose cells within 30 min of preparation for appropriate response from cells. This means, we may not observe same respones between fresh CSE and with −20 °C or −80 °C stored CSE for longer time. Thirdly, the project cost becomes expensive as we use fresh CSE and discard any leftover and we also need a separate cell culture incubator for only CSE study as the incubator smells of cigarette after repeated use and not suitable for growing normal cells (other than CSE related project).

## Conclusions

Our study clearly elucidated the many benefits of the nanotechnology-based approach in formulating ZER into LCNs such as having potent *in vitro* anti-inflammatory, antioxidant and antisenescence activity as well as the ability in slowing down the progression of airway inflammation as observed in RAW264.7 and BCi-NS1.1 cell lines. The anti-inflammatory action of ZER-LCNs was mainly observed in the inhibition of *IL-1β*, *IL-*6 and *TNF-α* gene expression as well as the reduced production of NO. The antioxidant activity was shown by the reduction and inhibition of total cellular ROS and the gene regulation of *GPX-2* and *GCLC* whereas the antisenescence was observed in the reduction of mainly *CDKN2A* gene expression. Hence, encapsulation of ZER in LCNs can potentially provide a new therapeutic avenue for validation of its role in the management of COPD through further extensive translational research/experimental models, including clinical study.

## Data Availability

Inquiries can be directed to the corresponding author.
